# Pedagogy with babies: perspectives of eight nursery managers

**DOI:** 10.1080/03004430.2015.1028399

**Published:** 2015-11-16

**Authors:** Peter Elfer, Jools Page

**Affiliations:** ^a^ School of Education, University of Roehampton, Grove House 214, Froebel College, Roehampton Lane, LondonSW15 5PJ, UK; ^b^ School of Education, University of Sheffield, 388 Glossop Road, SheffieldS10 2JA, UK

**Keywords:** pedagogy, babies, nursery, values, beliefs

## Abstract

The last 30 years have seen a significant increase in babies attending nursery, with corresponding questions about the aims and organisation of practice. Research broadly agrees on the importance of emotionally consistent, sensitive and responsive interactions between staff and babies. Policy objectives for nursery and expectations of parents and staff give rise to different and sometimes conflicting aims for such interactions; for example attachments to staff, peer interactions or early learning. Research shows marked variations of pedagogy aims and organisation with babies in nurseries in different national and cultural contexts. It also demonstrates variation between nurseries in similar contexts and between staff in their beliefs and values about work with babies. This paper reports on an exploratory study of the beliefs, aspirations and approaches of eight managers concerning pedagogy with babies in two similar English local authorities. These managers spoke of the importance of being responsive to the concerns and priorities of parents, whilst being sensitive to the demands of the work on their staff. The main finding was of the contradictions and confusions managers felt were inherent in the work, arising from both conflicting policy objectives and personal beliefs and aspirations; sometimes their own and sometimes those of individual staff and parents. Urban, Vandenbroeck, Van Laere, Lazzari, and Peeters' [(2012). Towards competent systems in early childhood education and care. Implications for policy and practice. *European Journal of Education*, *47*(4), 508–526.] concept of the ‘competent system’ is used to recommend a grounded approach to the development of a more culturally, socially and individually responsive pedagogy with babies than appears to exist at present.

## Introduction

### Background

The last 30 years have seen a significant increase, particularly in industrialised societies, in how babies are brought up from primarily within families to a combination of family and nursery (Organisation for Economic Cooperation and Development [OECD], 2012). There are variations in the extent of this shift, for example, between the USA, with relatively limited parental leave arrangements (Schore, 2014), and the Scandinavian countries, where parental leave is more generous (OECD, 2012). This trend has been referred to as ‘a minor social revolution’ (Brooker, 2010, p. 194).

This extension in the role of nurseries to include the daily care of babies is relatively new compared with the long history of nursery provision for three- and four-year-olds. It has occurred in response to competing and diverse policy objectives. These have included enabling the participation of both men and women in the paid labour market, reducing the socio-demographic gap in educational attainment and strengthening family support (Brehony & Nawrotzki, 2010). Alongside these different policy objectives there is a highly contested discussion of how the baby is best conceptualised as a person, as vulnerable and dependent, or as competent and with agency (Kalliala, 2014).

### Variations in pedagogic practice with babies

There are marked differences of pedagogy for babies between nurseries in different national and cultural contexts (Rayna, 2004). One might expect that for nurseries operating within the same policy context, perspectives on pedagogic practice would be similar and that there would be consistency between staff views and actual practice. Brebner, Hammon, Schaumloffel, and Lind (2015) did indeed find such consistency:

Early childhood educators have a strong belief that their role is to meet the physical, emotional and educational needs of the children … and they use their relationships with the children as a tool to help them achieve this. (p. 16)

This is an important and encouraging finding. Drugli and Undheim (2012), Elfer (2009), Page and Elfer (2013) have found that practice may significantly depart from the stated beliefs and aspirations of the staff. Brebner et al*.* (2015, p. 3) have pointed out there is a need for further work that includes interviews with staff and direct observations of their practice, so that triangulation between the two can occur. We would fully support this but also think there is a case to be made to interview managers and staff separately from observations of their practice.

### Aims of the research

The gap between beliefs and aspirations and observed practice is sometimes caused by lack of resources, for example too few staff (Datler, Datler, & Funder, 2010). There is evidence that the structural resources for emotionally responsive interactions in nursery are a pre-condition, not sufficient on their own, and there is a need to attend to the internal resources of the individual practitioner:

 … .early childhood teachers find they are charged with the task of establishing and maintaining an emotional intersubjectivity with several children at one time … .regardless of their own inner resources or ability to do so'. (Brennan, 2014, p. 289)

Brennan argues:

 … .to develop methodologies that will support investigation of the internal processes and experiences which promote secure adult-infant attachment, but with the adult's needs in focus. (2014, p. 289)

Our aim is to respond to this endeavour through an exploratory study focussing on one group of adults in the nursery community, the nursery managers. We acknowledge that work is needed to deepen understanding of interactions in nursery from the point of view of all stakeholders including parents, staff, managers and not least the babies themselves. Some of that work has been started already, in relation to parents (Elfer, 2009, 2014b), staff (Osgood, 2006; Powell & Goouch, 2012) and under threes (Elfer, 2006). Our focus here is on managers, because of their particular influence in the way any given set of policy and procedural requirements may be implemented (Leach, 2009, p. 195).

### Approach of the research

We agree with Brebner et al*.* (2015) on the importance of research investigating the factors that influence how adults’ views on working with babies in nursery translate into practice and where research designs must therefore include direct observations of practice. However, we also argue that there is a need for a complementary line of research where the emphasis is on enabling nursery staff to reflect more openly on the values and beliefs that underpin their views on work with babies. For research taking this focus, the inclusion of direct observations of practice alongside interviews may be unhelpful.

Here and in the literature review, we highlight the anxiety and ambivalence many staff working with babies appear to feel which may be a significant factor in the extent to which stated beliefs and aspirations translate into practice. This anxiety includes apprehension about parents’ resentment if babies become too attached to a particular staff member (Hopkins, 1988) and about babies’ dependency and the distress this may cause if attachments become too strong (Elfer, 2006, 2009). There is also a perceived unease about behaving inappropriately from a child protection point of view (Piper & Smith, 2003; Sikes & Piper, 2010).

Staff working with babies bring to their work a depth of personal feeling and involvement, alongside professional beliefs values (Manning-Morton, 2006; Osgood, 2004; Elfer, 2006, 2009, 2012; Page & Elfer, 2013).Our research has also demonstrated that the personal beliefs of staff about the value of nursery for babies, especially full time, is sometimes in conflict with their choice to carry out this work (Elfer, 2009).

Some staff may be prepared to explore these conflicts in interview, but for others our experience has been that it is not so straightforward to talk about beliefs and values when practice may also feel under scrutiny (Elfer, 2007, 2012; Page & Elfer 2013). It may be easier to facilitate trusting research encounters if the staff member's practice is not to be subsequently observed in detail.

### Terminology

Pedagogy: We have drawn on Dalli et al*.* (2011) in taking pedagogy to refer to a holistic approach to the care and learning of others, underpinned by a combination of ‘skills, knowledge, dispositions and associated strategies’ (p. 65).

Nursery: There is a wide variety of types of publicly regulated nursery provision for babies and young children, nationally and internationally. Concise terminology is problematic because it masks major differences of aims, ethos and context, whilst more descriptive terminology can be cumbersome. We have therefore used the term ‘nursery’ to refer to regulated provision for babies in non-domestic premises.

Baby: The more formal term for a baby under 12 months is ‘infant’ but we refer to ‘baby’ meaning under 12 months.

## Literature review

Review of the research literature shows at least three key contested areas of debate that are a context to research on pedagogic work with babies:

- The principle of babies’ non-familial care and the possible negative impact on long-term development.
- Multiple policy objectives and conflicts in the aims and organisation of nursery provision.
- Opposing conceptions of the baby as essentially ‘fragile’ or ‘resilient and competent'.

### The principle of babies’ non-familial care

Research on the possible negative impact of non-familial care on babies and young children has been extensive (McGurk, Caplan, Hennessy, & Moss, 1993, p. 11). These researchers argued that the evidence did not support ‘monomatry’ (the principle ‘that children should be reared by a single mother figure’) as a ‘privileged form of child care’ (McGurk et al., 1993, p. 19) and that meant non-familial care should be automatically regarded as harmful. This argument was supported by Rutter (2002) in a further review of evidence. In the USA, some evidence was found of the negative impact for babies experiencing long hours of low-quality nursery; with ‘low quality’ related to high levels of staff turnover and lack of sensitive responsive care (Belsky et al*.*, 2007). This concern about negative impact has been reinforced in further work reviewed by Schore (2014, p. 53).

It does seem that there are quite deeply held personal views about the value of nursery in the lives of babies and families (Karen, 1994). If instinctively some nursery staff feel critical or anxious about the impact of full time nursery on babies, there is an important question about how this might influence their pedagogic practice.

### Multiple nursery policy objectives

These include enabling the participation of both men and women in the paid labour market, reducing the gap in educational attainment for children considered to be disadvantaged, strengthening family support and improving social inclusion (Brehony & Nawrotzki, 2010). Different policy objectives give rise to different pedagogic approaches. One approach has been to see nursery as ideally organised so as to replicate aspects of family life. The English early years curriculum requires every baby to have a ‘key person’ who will facilitate a ‘settled relationship’ (Department for Education [DfE, 2014], p. 21). There is now a widespread emphasis internationally on early years policy with regard to the importance of attachment in nursery (Page & Elfer, 2013). However, Dahlberg, Moss, and Pence (1999) argue nursery:

 … .is not to be understood as a substitute home. Young children – both under three and over three years of age – are seen as able to manage, and indeed to desire and thrive on relationships with small groups of other children and adults, without risking either their own wellbeing or their relationship with their parents. (p. 81)

Another pedagogic approach has been to see nursery as conceptualised as school, with a focus on the organisation of learning. From 2002, babies have been included in the English national early years curriculum (DfE, 2014). It has been argued that regulation by the Office for Standards in Education (Ofsted) has reinforced an over-emphasis on narrow educational aims within nursery organisation and practice (House, 2011) which has led to the denigration of work with under threes (McDowall Clark & Bayliss, 2012). A third approach has been to emphasise the importance of peer interaction in which babies have an opportunity to realise a social life separate to that of the family, developing social interactions with peers and in groups (Dahlberg et al*.*, 1999, p. 82; Degotardi & Pearson, 2009; Dencik, 1989).

### The baby as ‘fragile’ or ‘resilient and competent’

Kalliala (2011) states that:

When we want to examine the adult role we have to start from the child. What children are like – or what we think they should be like … . (p. 238)

Underpinning these opposing conceptions is a broader discussion of the social position of the young child who is no longer growing up within the primary socialising context of the family but, in the case of babies in nursery, living a life of ‘dual socialisation’ in home and nursery (Dencik, 1989, p. 167). Dencik acknowledges the reality of this new situation for many young children, suggesting it will result in:

 … .a different sort of adult … instead of anxious, cautious, and conscience stricken adults, a generation may be growing up which is less willing to accept things as they are more open-minded, outspoken, and perhaps a good deal less conscientious. (p. 176)

This outcome has been interpreted by some as a polarisation between the old view of the child as ‘fragile’ and the new view of the child as ‘resilient and competent’ (Kalliala, 2011, p. 238). Traditionally, the emotionally ‘vulnerable’ view of the child has rested on attachment theory (Bowlby, 1988). The ‘competent’ view has drawn on the work of Trevarthen (2005) who has shown the rich capacities and intrinsic motivations of babies for inter-subjective behaviours. Kalliala (2011) challenges the argument that the view of the ‘fragile’ baby has dominated pedagogy whilst the view of the ‘competent’ baby has been lost but asks how these contrasting views can be combined (p. 239).

The view of the baby that is epitomised by personal experience or professional training, and the policy context within which they work, seems likely to influence how their pedagogy is shaped, consciously or not. We think attachment is key here. The avoidance of attachments in nursery may be evaluated as facilitating the baby's competencies through encouraging independent explorations and peer interactions whilst close attachments may be seen as encouraging security (Elfer, 2006).

Brebner et al*.* (2015) argue that nurseries ‘can play an important roll in attachment relationships’ (p. 2). We would not disagree with this. Indeed, Page (2011, 2014) has drawn attention to the desire of mothers that their babies should experience ‘professional love’ at the nursery. Such a general call for attachment relationships leaves a large question about what sort of attachments and for what purpose. When thought about away from the immediate pressures from the immediate pressures of the daily work and its regulatory expectations, it seems to us that the views of managers regarding this question and their underlying feelings and beliefs about what they think the baby is like are vitally important to explore.

## Methodology

### Recruitment of nursery managers

To meet the research aims, we recruited eight nursery managers from two local authorities (LAs). The sample was small enough to spread the available fieldwork time to ensure up to two hours for each interview, and large enough to ensure inclusion of nurseries with charitable and commercial status and located in affluent and poorer catchment areas.

The LAs were chosen partly because of their similarity (political administration, socio-demographic profile, charitable and commercial nursery mix and central Advisory Team (AT)) and partly because of our good relationship with these authorities based on prior research work.

Many LAs in England face severe financial constraints and the imposition of competitive tendering for their directly provided services, including ATs. Obtaining agreement for the research was therefore likely to be more difficult because of the time demands it imposed and partly in case negative aspects of services were exposed. The strategy of the research was to be particularly attentive to promoting trust in the researcher–participant relationship in order to enable the managers to speak as openly as possible about their pedagogic values and beliefs. Our good working relationship with the LAs enabled their ATs to be reassuring to their senior team in seeking permission for the research to take place as well as to the nursery managers. We wanted to reassure both groups about our commitment to better understand managers’ perspectives rather than to evaluate their practice.

Written information was given to the ATs about the research and its aims. Nurseries who fitted the criteria for the research (catered for babies and were open all day) were informed about the project and invited to put their names forward if they were willing to participate. From those expressing an interest, a provisional list of possible managers to be approached was compiled. These managers were contacted by e-mail, giving further information about the research, along with a follow-up phone call if requested but again emphasising the exploratory aims of the study.

### Interview approach

We designed an interview approach that was as sensitive as possible to any anxiety each manager may feel in discussing openly their values and beliefs in relation to pedagogic work with babies. In the Introduction, we referred to our experience that it is not always straightforward for nursery staff to talk about the values and beliefs which they consider underpins their practice (Elfer & Dearnley, 2007; Elfer, 2012; Elfer, 2013). We think there are at least three possible sources of this.

First, our professional development work with nursery staff has shown that sometimes staff draw on their own upbringing at home or within nursery and that this has strongly influenced their current beliefs and practices. These staff are often reluctant to disclose these personal sources of influence for fear that they will be criticised as unprofessional (Colley, 2006).

Second, Bain and Barnett (1986) and Hopkins (1988) showed that staff's initial disparagement of nursery attachments was expressed as a professional judgement that such attachments made some children over-dependent and some parents resentful (Hopkins, 1988, p. 102).

Third, we have each published research papers and professional development materials and spoken at conferences. It seemed likely that some managers would see us as ‘*the* experts’ making them reluctant to be open and confident about expressing their own beliefs and experiences.

We wanted therefore to develop research interactions with managers that enabled them to explore their beliefs about pedagogic work with babies in as uninhibited a way as possible. Here, we have been interested in the contribution of psycho-social studies as:

 … informing the development of new methodologies in the social sciences, including … the development of psychoanalytic ethnography/fieldwork and attention to transference-countertransference dynamics in the research process. (Clarke & Hoggett, 2009, p. 2)

Psychoanalytically informed interviews are underpinned by the concept of the defended subject and the notion that some individual experience may provoke too much anxiety to share openly with others or possibly even to be conscious of oneself. Their aim is to:

 … .go beyond the intentional narratives that are in danger of only revealing what interviewees consciously wish to know or show about themselves. (Hollway & Jefferson, 2013)

We firmly distinguish between probing intrusively into views that managers may wish to remain private and being open to views that managers may be hesitant to express in case of criticism but nonetheless wish to convey. Powell and Goouch (2012) have shown how sensitive nursery staff may be to such feelings of criticism or disapproval. Hopkins (1988) sought to minimise this by expressing interest in the origins of her participants’ views and asking them to illustrate issues where possible with examples from their practice. This, the minimal use of probing questions and sensitive attention to emotional atmospheres, seemed to facilitate deeper reflection and exploration (p. 108).

Each researcher conducted four interviews. Participants willingly consented to interviews being audio recorded in the knowledge that they could request the device to be switched off at any time. We explained that we were interested in their overall views on nursery work with babies and would like to hear about anything they considered important. Our interview approach was to encourage participants to develop or give examples of an idea or experience they had introduced to elaborate meanings in this way rather than through direct questioning.

### Framework for analysis

Immediately after the interviews each researcher recorded the main impressions and feelings evoked in themselves. The aim was that this should be as much as possible an unedited and free flowing reflexive record. These records were then discussed to deepen understanding of the interview itself and the influence of researcher subjectivity. Recent innovative work using shared reflexive capacity to deepen understanding of data has been encouraging (Elliott, Ryan, & Hollway, 2012; Epstein, Fahey, & Kenway, 2013). Two weeks after each interview, managers were invited to send by e-mail any further thoughts and reflections that may have occurred to them; four did so.

In analysing these data, we chose not to use analytic software because of its limitations in sensitivity (MacMillan, 2005). Rather, an initial set of three to five main themes was compiled by each researcher by repeated listening to the whole interview. These themes were checked by scrutinising line by line how sections of the interview challenged or supported a theme as described. In this way, it was possible to change, add and refine themes, underpinning their validity by illustrating them with rich sections of interview.

Before seeing this thematic summary, we swapped and reviewed each set of four audios and reached an individual assessment of themes. Emerging differences facilitated our critical discussion about how these had arisen so that further reflection about possible meanings in the data could occur.

The analytic framework is, of course, essentially interpretative. We have confidence though that it has enabled us to convey features of the beliefs, certainties and uncertainties of the participants in relation to managing pedagogic work with babies. The interpretations are ours and they apply only to these managers in these particular nursery contexts. Subsequent work using a similar methodology but in different contexts may be able to support or challenge the wider applicability of these interpretations.

## Findings and commentary

### Organisational context

Table 1 presents data on the organisational context of each of the nurseries.

**Table 1.** The organisational context of each nursery.

	Total number of places available for children aged 0–5 years	Single or nursery chain?	Youngest age range and number	Age range of babies/young children in one room	Take babies from (months)	Hours of opening	Respondents title and highest level of qualification^a^	Baby staff qualifications^a^	Ofsted grading^b^
		A = single;							
		B = < 5 nurseries;							
		C = 5 + nurseries							
M1	102	C	32 × 3–24 m	3–12 m	3	7.30–6.00	Manager: Level 4	1 × level 4	Outstanding
						51 weeks of year		4 × level 3	
								1 × level 2	
M2	26		12 × 3–24 m	3–48 m	8	8.00–5.00	Manager: Level 4	1 × level 5	Good
						Term time only	Senior B R Practitioner: BA (Hons)	2 × level 3	
M3	57		12 × 3–12 m	3–24 m	3	7.15–6.30	Manager: BA EYS	2 × level 3	Outstanding
						51 weeks of year		2 × level 2	
M4	174		12 × 3–12 m	3–12 m	3	7.30–6.30	Owner/Manager: BA (Hons) EYS with EYPS	2 × level 3	Outstanding
						51 weeks of year		3 × level 2	
M5	80	C	12 × 3–15 m	3–15 m	3	7.30–6.30	Principal – BA Hons (Early Ed) + NNEB	1 × level 3	Good
								1 × level 2	
M6	77	A	21 × 3–24 m	3–15 m	3	7.30–6.30	Manager	3 × level 3	Good
						51 weeks of year	Foundation degree in EYs + BA Hons (Leadership)	1 × level 2	
M7	46	A	6 × 3 m–24 m	3 m–12 m	3	8.00–5.30	Manager Level 6 with EYPS	2 × level 3	Good
M8	98	B	20 × 3 m–15 m	3 m–15 m	6	7.30–6.30	Manager /joint owner – BA with EYPS	2 × level 3	Good
								2 × level 2	

^a^EYPS = Early Years Professional Status was a qualification introduced by the UK government in 2007 intended to be broadly equivalent to qualified teacher status. It has now been phased out and is being replaced by Early Years Teacher Status.

^b^Ofsted is the UK national inspection agency. It inspects early years and day care provision awarding four categories of grade (outstanding; good; requires improvement; inadequate).

We report the findings and comment on them in two sections, first on what managers said from the perspective of the ‘fragile versus competent’ debate; second from the perspective of pedagogic practice.

## Babies’ fragility and competence in the narratives of the managers

### Interview extracts

Did these eight managers see the baby as the ‘fragile novice child or the resilient, competent child’ (Kalliala, 2014)?

Whilst each narrative was highly individual, they were similar in their fluctuating literal meaning and emotional tone, sometimes expressing passionate conviction and sometimes uncertainty, even contradiction. In relation to ‘fragility versus competence’, the managers each made reference to the presence of both characteristics:

I think, for me, the babies are the most vulnerable age group we ever look after … . the babies depend so much on the right key person, the right adult. For me, personally, there's no better place for a baby than to be at home … (M1)

 … to help them cope with being able to sit for a little while … that's really traumatic for some babies to be put down and although we wouldn't put them down for very long, that's something you're helping them to move forwards with … . (M7)

### Commentary

It was clear that none of the managers simply located the baby as either ‘fragile’ or ‘competent’. This is perhaps unsurprising as these managers are immersed in daily intimate care of babies as real individuals and subject to the powerful feelings of protectiveness (fragility) and pride (competence) that babies can evoke.

The managers spoke of the baby in the context of relationships with the ‘right adult’, those who are consistent, sensitive and responsive. Their view is resonant with Winnicott's (1960) famous assertion that the baby can only be understood in the context of relationships with others (p. 99). It focuses attention on a central question of pedagogy of exactly what kind of ‘relationships with others’ matter. We have argued elsewhere that much early international years policy calls for attachment relationships (Page & Elfer, 2013).

The argument put forward by Dahlberg et al. (1999, p. 81) is that the emphasis on nursery attachments is unnecessary. They argue that it emphasises the view of the baby as ‘fragile’, unable to engage in interactions outside of the primarily dyadic protective role of maternal care. They instead emphasise competence:

Young children – both under and over three years of age – are seen as able to manage, and indeed to desire and thrive on relationships with small groups of other children and adults. (p. 81)

Kalliala (2011) objects to this conceptual view of competence asking ‘in what way is the child competent?’ (p. 239). She cites Kampman (2004) in stating that:

the idea of the competent child … . is neither based on daily observations nor research findings which might show children are more competent than believed. (p. 239)

Findings from neuroscience have converged with those of development psychology and psychoanalytic theory in stating the importance of adults containing babies’ emotions:

When a baby is upset a carer often emphatically shows they understand by making noises rather like the infant's … a slightly exaggerated reflection of an infant's feelings, not quite hamming it up but not quite real, conveying a sense of an emotionally attuned mind alongside them, bearing their feelings and reflecting them back … something that … . Bion (1963) called emotional containment. (Music, 2011, p. 29)

In order for nurseries to provide this containment, it may be necessary, indeed inevitable, that attachments are formed and sensitively mirror those at home. It also seems clear that it is not helpful to conflate nursery and home attachments as seems to happen in some statements about making nursery like home:

I guess quality care for infants and toddlers is as much as possible caring about the child and the family. The ‘homing’ environment … Carrying on what they do at home as much as possible here. (Brownlee, Berthelsen, & Segaran, 2009, p. 462)

Home and nursery attachments cannot be the same as the adults involved come to the interaction in completely different ways and for different purposes. The home attachment is likely to be a deeply experienced cultural practice. The nursery attachment can be seen as responsive to fragility but also facilitative of competency. It need not be understood as indicating a view of the baby only as ‘fragile’.

## Pedagogy in the managers’ narratives of practice

Here the data could be organised as four dimensions.

### Attention to babies’ emotional experience

#### Interview extracts

Every morning … he looks for his key person (KP); when he hears her voice he turns his head towards it and beams and holds out his arms to be welcomed by her; he pulls back towards his mum momentarily then … shifts his body over to her and nuzzles into her shoulder … (M2)

The staff take turns to changes nappies doing all 12 at their ‘turn’ … . I used to do 24! It's (the KP) all a bit over the top … . and even with a KP and a buddy (back up), it's too much’ (M8)

These extracts represent the extremes of how managers differed in the way they spoke of nursery attachments, the first seemingly favourable, the second somewhat dismissive. Mainly though, managers encouraged attachments in principle whilst regulating them in practice, for example where they were seen as motivated by the staff member's own needs, where past experiences were too painful or where there was anxiety about parents’ resentment:

 … .in that case the attachment was wrong because she wanted the children to fulfil what she didn't have. (M1)

(on the extended leave of a member of staff to whom a baby was strongly attached) … it was almost like you had to resettle that child and parent … . it was really upsetting (M6)

 … some parents don't want you to have too close a relationship ‘this is my baby mine! (M6)

Alongside concern about limiting emotion, there were references to love:

 … love is a strong word. I adore some of them. I think love is an emotion that's very personal isn't it? Love is almost non-transferrable and you have to transfer your affection … (M2)

 … having someone you trust and you love and can go to for a cuddle (M3)

Her vision (the owner's) was to provide a place for children to come for education and a place to be loved and to be safe … . (M7)

Oh you do love them all … . but you would never use that word. (M8)

#### Commentary

Taking the two quotations at the beginning of this section, why should it be that two managers, working in similar authorities, in the same national context and where attachment is an underpinning principle in the common regulatory framework (DfE, 2014) see attachments so differently?

One explanation is that referred to earlier in this paper set out by Bain and Barnett (1986) and Hopkins (1988). Their argument was that attachments may be dismissed by staff because of prior experience of how painful these could prove in practice, for example when children became over-dependent, the experience of loss when children or staff moved on, or when parents felt rivalry with staff over their child's affections.

More recent work (Elfer, 2014b) has shown that whilst some of these anxieties remain, others have been replaced by new anxieties, for example increased unease about relations between children and adults generally and suspicion about any physical holding or touching of children by adults (Piper & Smith, 2003; Sikes & Piper, 2010).

It is interesting that four managers (including M8) should use the word ‘love’ in describing their feelings towards the children and this does seem important to some parents (Page, 2011). These four quotations also indicate some anxiety about the word. If the personal demands on nursery staff of being closely engaged with specific babies provokes anxiety, it seems likely that this will lead some to avoid attachment interactions in practice. In this context it seems hard to imagine how practitioners will engage in the detailed pedagogic reflection necessary to review nursery attachments and their own emotional investments in them.

Most early years practitioners, along with others in the ‘helping professions’ have an image of themselves as giving caring people with ambition to love and be of service … when the child runs in with a hug or the parent is grateful there is satisfaction. The problem with this idyllic picture is that it is not real; children also reject practitioners, and parents criticise. (Manning-Morton, 2006, p. 48)

In addressing the complexities of nursery attachments, their aims and deployment, we support the argument of Brennan (2014, p. 289) that the focus of concern must broaden from being on the baby to being on the baby–practitioner interaction. Fortunately, there does seem to be a turn in the research literature from a focus on cognition and learning to giving more attention to the affective dimensions of nursery practice (Brennan, 2014; Osgood, 2004; Taggart, 2011; Page & Elfer, 2013) and the support staff may need if they are to critically engage with the personal as well as professional facets of their interactions with babies.

### Planning and facilitating babies’ exploration

#### Interview extracts

 … .well they're enjoying that now why am I waiting till next week to carry out that activity?’ So you almost do it like the next day or arrange things as soon as possible (M2)

Does it matter that we planned to do something else? No! And I think that if they came to me and say we had planned to do this today and we didn't do it because … ..I certainly wouldn't criticise them … . (M6)

#### Commentary

All the managers spoke of a commitment to planning based on observations and the need to abandon planned activities when the baby's responses dictated something else. They also described, usually implicitly, their belief in each baby's agency, when they referred to the importance of planning in the context of an individual baby's competencies and interests.

These data raise an important issue in relation to the principle of planning in pedagogy for babies. The notion of observation and planning is central in some pedagogic guidance:

….the skilful practitioner will, in planning for children’s learning, give due care to providing developmentally appropriate and meaningful play experiences for all children. (Scottish Government, 2014, p. 48)

Babies like routines and predictability but their mood states and receptivity fluctuates rapidly. What is the balance to be drawn between planned interactions based on observations as suggested in the above guidance and the attuned spontaneity of sensitive and responsive interactions? Dalli et al. highlight the difference:

 … in countries such as Australia and America … ..Pedagogical strategies … . emphasised provocation and the strategic promotion of scientific concepts … were therefore more directed and focussed based on teacher observations of children at play … . rather than the socio-cultural orientations evident in New Zealand practice. (2012, p. 73)

The English early years curriculum framework, like its Scottish counterpart, emphasises observation and planning (DfE, 2014). Yet the managers seem to be advocating an approach more in keeping with the socio-cultural tradition of New Zealand practice. Trevarthen and Malloch (2002) also write of the socio-cultural roots of baby's early learning and the role of spontaneity:

Babies are totally naïve about music culture yet they hear the sounds of music well … .And how can we explain how adults and siblings spontaneously make musical sounds that attract babies – how they can talk baby talk and sing nursery songs … .? (p 17)

### Working with families

#### Interview extracts

 … .a lot of the time it's sort of a distraction technique … ‘oh we've done this today’ so taking away from the fact that the child is sort of trying to wriggle out of their (the parents) arms and go back (to the keyperson) – there's lots of communication that goes on to distract the parent from thinking actually ‘mm, they don't want to be with me’ (M2)

 … .the staff, they talk to parents, they listen to parents and they take on board parental comments, so that's kind of the ethos of the whole nursery, we do listen to parents … (M6)

We had somebody come, it's an extreme example and wanted their baby to start by the end of the week … what about feeding does she take a bottle? Oh no, she is breast fed … I thought you could help me with that … (M7)

#### Commentary

Sensitivity to parents’ position as customers, able to take their child and their fees elsewhere, was evident in the managers’ relations with parents, although it was more acute for the managers of commercial nurseries. For one manager, the pressure experienced from parents was intense. It took the form of continual pressure to open for longer hours, (the nursery already offered an 11-hour day), resentment if babies were unwell and parents had to be called from work (the nursery had introduced ‘deep cleans’ to reduce the risk of cross infections between children), and relentless pursuit of improved parent evaluation ratings. This stressful impact is in line with Osgood's (2004) findings.

Intertwined in relations with parents as customers were relations with parents as parents, emotionally sensitive to their own experience of nursery and their baby's relationships there. Managers were concerned to reduce parents’ anxiety, guilt or hostility. For many parents, this demanded little more than the ordinary sensitivity of an experienced practitioner. For some parents, the emotional work was complex as for M7 above. In this case, and there were other examples too, the parents seemed to invest the nursery with a form of omnipotence. We wondered if this might be a defence against parents’ possible guilty feelings, however unwarranted, of leaving their baby at nursery. If nursery can be constructed as ‘omnipotent and all capable’, it may relieve the parent to feel that nursery is ‘better’ than home.

The fundamental importance of partnership with parents is clear in research reviews (Dalli et al*.*, 2011; Mathers, Eisenstadt, Sylva, Soukakou, & Erkey-Stevens, 2014). Ahnert and Lamb (2003) have shown the importance of a partnership that enables close liaison between the two sets of adults in the baby's dual social worlds of family and nursery. It is the adults and not the baby who manages the task of mediating the baby's stressful experience as s/he moves back and forth between these social worlds.

Whilst this is important in principle, these managers’ narratives illustrate the emotional complexity in practice of establishing sensitive working relationships with parents. Parents also struggle with the realities of daily relationships with nursery staff (Leach et al*.*, 2006) and Brooker too has shown how relations between parents and staff are ‘fraught with opportunities for misunderstandings’ (Brooker, 2010, p. 194).

What is surprising, given that the importance of partnerships with parents is hardly a new finding, is that only very limited attention has been paid in the research literature to the complex work of facilitating these. Even these eight managers seemed to see their complex and sensitive work with parents in the ‘taken for granted’ way identified by Taggart (2011).

### Managers’ relationships with staff

#### Interview extracts

 … you have to be confident on the outside because you need respect from staff, I can't go into a room if there's an issue and go ‘ooohh I don't know what to do!’, I panic, they panic, the children panic, the parents panic. (M1)

They might come and say oh this has gone wrong, that's gone wrong, they're really grizzly and … .OK! take a step back … they have supervision sessions on a 1:1 basis every 6 wks; one of the questions I always ask is *can you reflect on something you have done – wow, that's why I do this.* (M6)

#### Commentary

These extracts illustrate the different ways in which the managers talked about supporting their staff and in particular, staff feelings of the work ‘going wrong’ illustrated by examples of many babies crying, upset parents or conflicts between staff. Analysis of the interviews showed the extent of stress and anxiety for staff and managers evoked partly by the demands of the babies and partly by the uncertainties and dilemmas of their practice. The managers conveyed clearly how the daily reality imposed by the market, the regulatory system and the contingencies of any organisational enterprise is often very different to the managers’ ideal. Osgood (2006) has theorised the management of this gap with the concept of ‘performativity’, evident in the extract from M1. This concept has considerable explanatory power.

However, managers’ responses show the need for a further way of theorising this beyond a ‘performance of confidence’. Our own work has shown how when staff are too anxious, they may also turn to rule-bound methods and over-reliance on procedural ways of working (Elfer 2007, 2012). Further, Page (2013) have shown the struggles of parents to manage compromises to their own ideals for the benefit of their child's happiness and well-being:

Ayesha explained … Lillian always changed Freddie's nappy on her lap and occasionally Ayesha noticed when she got home that his body suit was slightly soiled. Although she was upset the first time it happened Ayesha was also confident that Freddie had not been neglected and came to the conclusion it was both an oversight and a minor point. Ayesha recognised that Lillian's care was different to the way she did things but concluded it was a small price to pay for what she described as ‘the luxury of the close relationship between Freddie and Lillian'. (p. 555)

The management of levels of anxiety in staff is crucial. The extracts illustrate how thoughtful these managers were in responding to anxiety but M6 goes further in referring to ‘supervision’. This term has recently been introduced into English early years policy and is used in the context of a requirement that supervision should ‘foster a culture of mutual support, teamwork and continuous improvement, which encourages the confidential discussion of sensitive issues’ (DfE, 2014, para 3.21).

The introduction of this requirement coincides with recent calls in the literature for a turn towards systematic attention in research and practice to the impact on nursery staff of their work with children and families and, conversely, the impact of the emotional internal world of staff on their pedagogic practice (Brennan, 2014; Brooker, 2010; Brownlee et al., 2009). Our work on the development of Work Discussion groups (Elfer, 2012, 2014a) evaluates this approach as one model of supervision.

## Discussion

Our interviews were convincing in relation to how hard these managers worked and how much they achieved; the physical safety of the babies, attention to their well-being, empathy towards parents and support of their staff. They did all this in the context of pressure to maximise occupancy and minimise costs.

This considerable achievement of managers was all the more impressive because it was undertaken in a social and political context characterised partly by the low status of this work and partly by confusing and sometimes conflicting policy objectives and ‘approved’ practices.

Confusion lay first in fundamental uncertainties about the primary aims of nursery, for example whether the role of a nursery is an extension of family organised for attachment; an extension of school organised for learning or a different kind of social space to either family or school. There was also confusion in the meaning of being a ‘professional’ and how this fitted with the intimate, personal relationships that many staff instinctively felt babies needed.

The extent of these confusions emerging in the interviews was further underpinned by the data recorded in our immediate post-interview reflexive diaries:

He spoke confidently and positively about his passion for babies, for his practice and his aspirations for the nursery. He seemed quite nervous for most of the interview and I wondered if he felt ‘exposed’ and ‘vulnerable’ … . he ‘struggled’ when I asked him about the challenges of nursery practice. He looked decidedly uncomfortable and I wondered how long it would take for him to feel able to comment on his true feelings … (Extract of diary after M1 interview)

The interview itself felt flat, business-like and most of all, brisk. I had the strong feeling of ‘getting through’ it … .There was no sense of reflecting, mulling anything over … . Comments seemed like text books ones and she seemed reluctant to engage in any critique of nursery policy and practice, partly through her loyalty to the nursery company brand … (Extract of diary after M5 interview)

We understand these impressions of uncertainty as arising from managers’ work in a social context where extensive publicly regulated provision for the care of babies during the full time working week is relatively new compared with the long history of nursery provision for three- and four-year-olds. There does not appear to be a coherent policy approach to nursery aims and organisation for babies. If this is true, it is not surprising that managers struggle to integrate personal feelings and values, professional training and regulatory pressures, each pulling in different directions.

We are not arguing that managers can resolve the bigger anxieties and uncertainties that arise in rapidly evolving societies about changing patterns of social care of babies. What we propose is that provision for babies in nursery, for which there is clearly parental demand, would strongly benefit from the development of a much more coherent body of underpinning principle and practice.

The detailed findings from the interviews show how much of a resource these committed and experienced managers could bring to the development of such a body of principle and practice. The managers seemed to be trying to do this within their own staff groups, in different ways. It was outside the scope of our research but it seemed to us that those able to do it in more depth were those who were protected from the commercial priorities and pressures facing each nursery because they had a business manager/owner who took responsibility for these tasks.

In this respect, there is a resonance between the meaninglessness of conceptualising the baby as either ‘fragile or strong’ (much depending on the baby's relationships with others), and the meaningless of conceptualising the nursery as either ‘fragile or strong’ (much depending here too on relationships and context). Where a manager's self-confidence was strong (that is they were prepared to embrace uncertainty and had the courage to reflect openly, and with key others in their immediate environment), then the nursery appeared strengthened as a thinking, developing organisation.

Here we would make reference to the relevance of Urban, Vandenbroeck, Van Laere, Lazzari, and Peeters’ (2012) work on the ‘competent system’ with its four component dimensions:

- the competent individual (the capacity for insight and critical self-reflection);
- the competent institution;
- competent relations between settings;
- competent governance, (p. 516)

If we are seeing a sustained shift in social patterns for the upbringing of babies, from primarily within family networks to a combination of family and publicly regulated nursery, then the principles and underpinning theory of the public care of babies needs to be developed into a more coherent body of policy and practice. In our view, this cannot be done by a top-down, research-informed conventional training programme. Research matters, but theory and practice needs to evolve in a way that is much more sensitive to the wishes and expectations of families and communities. In this bottom-up approach, the ‘voices’ of the babies themselves also need to be heard. There are some models for doing this, for example in the form of ‘communities of practice’ (Wenger, 1998) and Work Discussion (Elfer, 2014a). This seems to us essential to develop in a ‘system that is competent’ but it is not sufficient. More attention is also needed to the role of local advisory services and the sensitivity of regulatory systems to capturing data on innovative processes and practices as well as compliance with statutory requirements.

## Conclusion

Our data have shown that from the perspective of these eight managers, each intimately involved in daily work with babies in nursery contexts, the theoretical discourse of the fragile versus competent baby is not persuasive. These managers know babies within the context of families, attachments and broader social interactions, not as isolated individuals. From this perspective, they see babies as both fragile and dependent, as well as having agency and autonomy.

Our data also contribute to a much needed mapping of pedagogy setting out four dimensions of practice, rooted in the daily work of managers supporting staff working with babies.

How much confidence can be invested in these data? It arises from a small sample of in-depth interviews with experienced managers in a small administrative area of a highly diverse nursery sector in one country. Despite this we consider these findings do have weight. These outcomes resonate with other emerging work on pedagogy with babies which embraces policy conflicts and confronts the uncertainties and dilemmas of developing local models of pedagogy that are informed by the voices of parents and babies themselves, as well as nursery professionals and local communities.

This study has two important findings arising from the implications for managers and their staff of doing this work in a social context characterised by ambivalence about the place of babies in nursery and the social policy aims of this provision. There is acute anxiety and uncertainty about managing the complexities of this work in a way that facilitates close emotional attachments for the babies, whilst also being sensitive to the proprietorial feelings of parents. These eight managers appeared to us to work extremely hard, but at considerable cost to them in the stressful demands of work that is poorly remunerated and of low status. More generally, the cost is the absence of any coherent body of theory and practice that was informed by the voices of babies, families and staff.

## Disclosure statement

No potential conflict of interest was reported by the authors.
